# The Use of Combined Hip Arthroscopy and Periacetabular Osteotomy for Hip Dysplasia Is Increasing and Has Low Complication Rates

**DOI:** 10.1016/j.asmr.2024.100929

**Published:** 2024-03-26

**Authors:** Juan Serna, Kira Furie, Stephanie E. Wong, Ishaan Swarup, Alan L. Zhang, Mohammad Diab

**Affiliations:** Department of Orthopaedic Surgery, University of California, San Francisco, San Francisco, California, U.S.A.

## Abstract

**Purpose:**

To analyze the annual use of hip arthroscopy (HA) and Bernese periacetabular osteotomy (PAO) for the treatment of hip dysplasia (HD), as well as postoperative outcomes, including ipsilateral reoperations.

**Methods:**

*International Classification of Diseases*, *Ninth* and *Tenth Revision*, codes were used to query the PearlDiver Mariner database from January 2010 through January 2022 to identify patients aged 10 to 59 years who had a presenting diagnosis of HD and subsequently underwent (1) HA; (2) PAO; or (3) combined HA and PAO (HA-PAO, defined as PAO on the same day or within 28 days after HA). We analyzed annual rates for each treatment, as well as rates of postoperative emergency visits, readmissions, and 5-year ipsilateral secondary operations (determined via Kaplan-Meier analysis).

**Results:**

There were 32,068 patients who underwent surgical treatment of HD. For HA, PAO, and HA-PAO, there were 29,700, 2,083, and 285 patients, respectively. All operations had the greatest percent-increase from 2015 to 2016. HA and HA-PAO peaked in 2021, whereas PAO peaked in 2019. For HA, PAO, and HA-PAO, most cases were performed in female patients and patients aged 30 to 49 years, 10 to 19 years, and 10 to 29 years, respectively. The 5-year incidence of ipsilateral secondary operations, which include revision HA, PAO, or conversion to total hip arthroplasty, was 9.2% (95% confidence interval 8.6%-9.8%) in the HA group and 6.5% (95% confidence interval 4.1%-8.8%) in the PAO group. Combining HA with PAO resulted in so few secondary operations that Kaplan-Meier analysis was infeasible. The PAO cohort had the greatest 30-day emergency visit and 90-day readmission rates, with infection as the most common cause for readmission.

**Conclusions:**

HA is more frequently performed than PAO for hip dysplasia. HA-PAO is increasing at the greatest rate, demonstrating fewer complications and reoperations.

**Level of Evidence:**

Level III, retrospective comparative trial.

Hip dysplasia (HD) represents an “abnormal formation” of the acetabulum primarily and the head of the femur secondarily. HD includes deformity, insufficiency or malalignment of the acetabulum, labrum tear, and chondral injury,^1^, ^2^, ^3^ as well as abnormal morphology of the proximal femur, including torsion and head-neck osteophyte.^4^, ^5^, ^6^, ^7^ HD without dislocation typically is silent in the first decade, when it is diagnosed principally by screening physical examination and imaging, such as ultrasonogram or radiography.^8^, ^9^, ^10^, ^11^ Patients begin to present in the second decade, with symptoms including groin pain, crepitus, signs of instability, and reduced motion.^12^^,^^13^

In the first 2 years of life, treatment of HD consists of bracing and closed versus open reduction under anesthesia as indicated.^14^^,^^15^ After 2 years of age, treatment includes osteotomy, as the hip is a bone-dependent joint that requires precise fit—any deviation from which will have a natural history of premature degeneration.^16^^,^^17^ Because the osseous deformity is considered the primary factor in HD, osteotomy alone traditionally has been performed for treatment based on the assumption that the soft tissues will be relieved of abnormal forces and will recover or become clinically silent.^18^, ^19^, ^20^ In the mature teenager and young adult with borderline HD, hip arthroscopy alone (HA) may be considered to address the soft-tissue consequences of osseous deformity—for instance, labral repair and chondral debridement to address labral tear and articular cartilage injury.^21^, ^22^, ^23^, ^24^ Recently, HA has been combined with Bernese periacetabular osteotomy (PAO) for comprehensive treatment of HD, based on several factors. First, there is a high rate of soft-tissue injury, which now can be directly and effectively treated, hence HA.^23^^,^^25^, ^26^, ^27^ Second, soft-tissue work will fail in the setting of substantial osseous deformity, hence PAO. Third, recognition and collaboration between orthopaedic subspecialities, from pediatric to sports to reconstruction, has allowed comprehensive treatment of all components of the dysplastic hip.^28^, ^29^, ^30^, ^31^ Given these 3 possible approaches to the treatment of HD, characterization of outcomes for each is important to aid orthopaedic surgeons in optimizing treatment for patients with this condition; this study thus seeks to add to the evidence-based approach for the surgical management of HD.

The purpose of this study was to analyze annual use of HA and Bernese PAO for the treatment of HD, as well as postoperative outcomes, including ipsilateral reoperations. We hypothesized that the rate of combined HA and PAO would be increased, with a low risk profile and improved postoperative outcomes reported.

## Methods

A retrospective cohort study was performed using the PearlDiver Mariner database (PearlDiver, Colorado Springs, CO).^32^ Mariner is an administrative database of commercial insurances, Medicare, Medicaid, and self-payor claims in the United States that comprises more than 161 million patients and longitudinally tracks individual diagnoses and procedures.^32^ This study used deidentified data and was exempt from institutional review board approval at our institution. The database was queried from January 2010 to January 2022.

*International Classification of Diseases*, *Ninth* and *Tenth Revision*, codes (ICD-9 and ICD-10) were used to identify patients who had a presenting diagnosis of HD^33^ and subsequently underwent (1) HA alone, (2) PAO alone, or (3) combined HA-PAO (see Appendix Table 1, available at www.arthroscopyjournal.org, for the diagnostic and procedural codes used as inclusion criteria). Patient ages were restricted to a minimum of 10 years, after which PAO becomes the dominant procedure for acetabular realignment, and maximum of 59 years, as total hip arthroplasty (THA) is the dominant procedure beyond this age. A combined procedure was defined as PAO on the same day or within 28 days after HA; these patients were excluded from the other 2 cohorts to define either HA or PAO. For patients undergoing HA, previous PAO was an exclusion criterion; conversely, for patients undergoing PAO, previous HA was an exclusion criterion. Other exclusion criteria were history of hip fracture, infection or neoplasm within 31 days preceding the operation, as well as any previous THA.^34^ In addition, patients undergoing other nonhip arthroplasty before or after index surgery were excluded because of the lack of ICD-9 and ICD-10 complication codes specific to the hip.

Data were collected for the cohorts both with and without regard to laterality using the laterality inherent in ICD-10 diagnostic codes. Nonlateralized cohorts were analyzed to determine annual numbers (through January 2022, so as to analyze full years only) in each surgical category (HA, PAO, HA-PAO). Data analyzed included procedure cases per year, as well as by age group and by biological sex. In addition, readmissions and emergency visits were analyzed using nonlateralized data; tracking time was expanded through April 2022 to encompass all event instances, since these data only required tracking for 90 and 30 days, respectively. Lateralized cohorts were analyzed for incidence of secondary operations. Because sample sizes less than 11 are not reported by PearlDiver to protect patient privacy, annual numbers of the HA-PAO cohort were estimated from the total sample size for 2011-2015.

Rates of postoperative emergency visits and readmissions were tracked only for those patients with 90 days of active enrollment after operation. For HA-PAO, tracking began after the latter procedure. Current Procedural Terminology (CPT) codes (CPT-99281, CPT-99282, CPT-99283, CPT-99284, CPT-99285, CPT-G0380, CPT-G0381, CPT-G0382, CPT-G0383, CPT-G0384) were used to assess rates of postoperative emergency visits within 30 days postprocedure.^34^ PearlDiver software provided both readmission numbers and reasons; only unique patient admissions within 90 days postprocedure were included in the calculation of readmission rates.

Secondary operations were defined as HA, PAO, or THA within 5 years of index procedure (Appendix Table 1). To ensure that the secondary operation occurred on the same side (ipsilateral), all cohorts were lateralized by linking CPT procedural codes for HA, PAO, or THA to left- or right-sided unilateral ICD-10 diagnosis codes only.^34^ Because laterality analysis is only possible using ICD-10 diagnosis codes, only patients with index procedures from 2015 and onward were included.

Cumulative incidences of ipsilateral secondary operations were determined via Kaplan-Meier survival analysis, which was performed using R statistical software, version 4.3.1, integrated with PearlDiver (R Project for Statistical Computing, Vienna, Austria).

## Results

**Fig 1:**
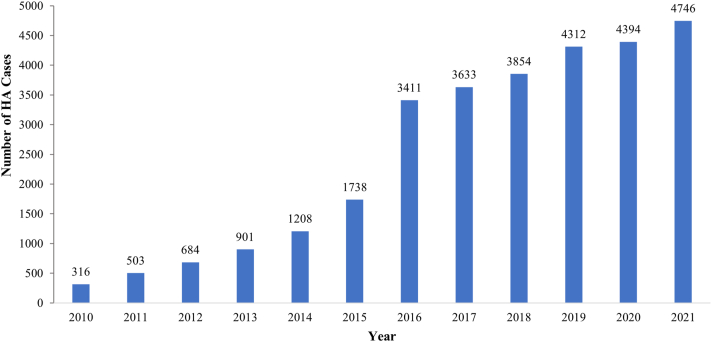
Yearly use of HA for the treatment of hip dysplasia. Note the continued increase. (HA, hip arthroscopy alone.)

**Fig 2:**
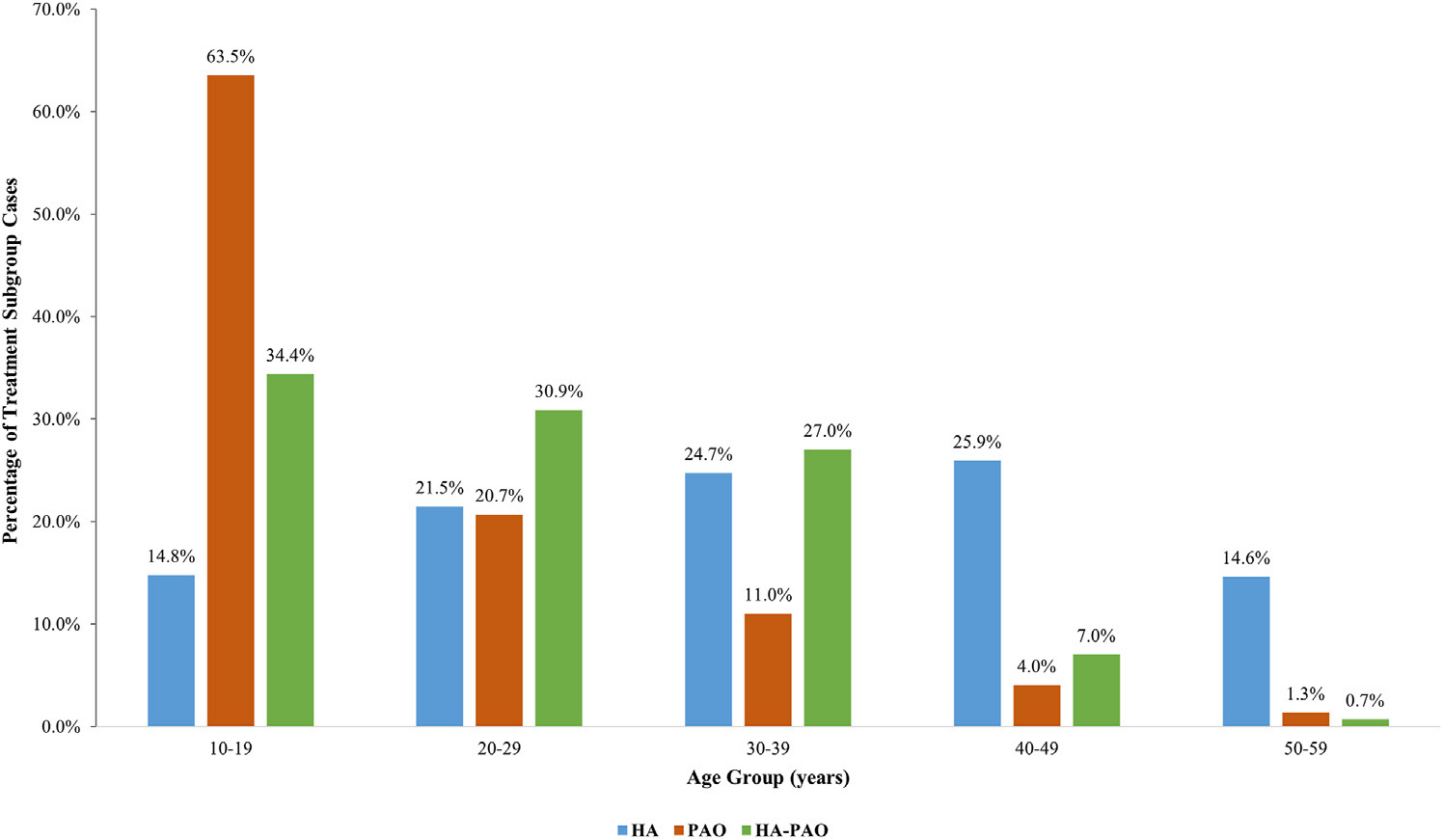
Age-group analysis for HA, PAO, and HA-PAO for the treatment of hip dysplasia. Note that the majority of patients (99%) in the 10-19 age group for HA were ages 15-19. (HA, hip arthroscopy alone; HA-PAO, combined hip arthroscopy and periacetabular osteotomy; PAO, periacetabular osteotomy alone.)

From January 2010 to January 2022, 32,068 patients with HD underwent surgical treatment. There were 29,700 cases of HA: the rate doubled from 2015 to 2016 and peaked at 4,746 cases in 2021 (Fig 1). Most were performed in patients aged 30-39 years (24.7%) and 40-49 years (25.9%) (Fig 2). Almost all cases (99%) in the 10- to 19-year age group were in patients aged 15-19 years (i.e., mature).

**Fig 3:**
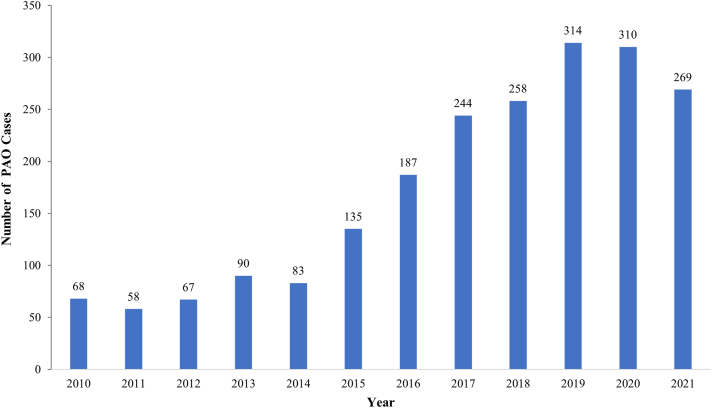
Yearly use of PAO for the treatment of hip dysplasia. Note the recent decline. (PAO, periacetabular osteotomy alone.)

There were 2,083 patients who underwent PAO: there was a 1.4× increase from 2015 to 2016, and a peak of 314 cases in 2019 (Fig 3). The majority of these (63.5%) were performed in patients aged 10 to 19 years, with only 1.3% performed in the 50- to 59-age group (Fig 2).

**Fig 4:**
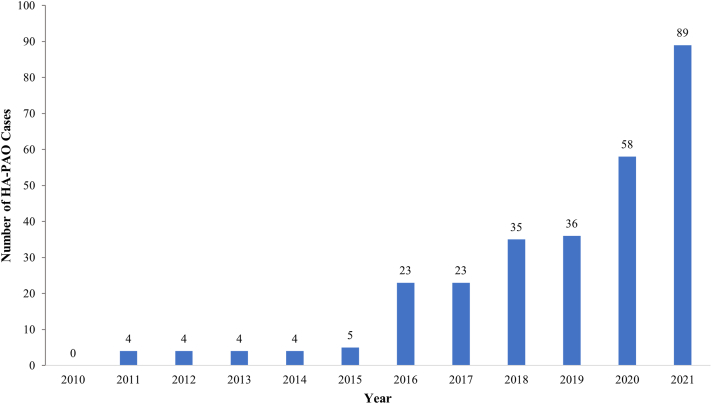
Yearly use of HA-PAO for the treatment of hip dysplasia. Note the continued and most rapid increase. (HA-PAO, combined hip arthroscopy and periacetabular osteotomy.)

**Fig 5:**
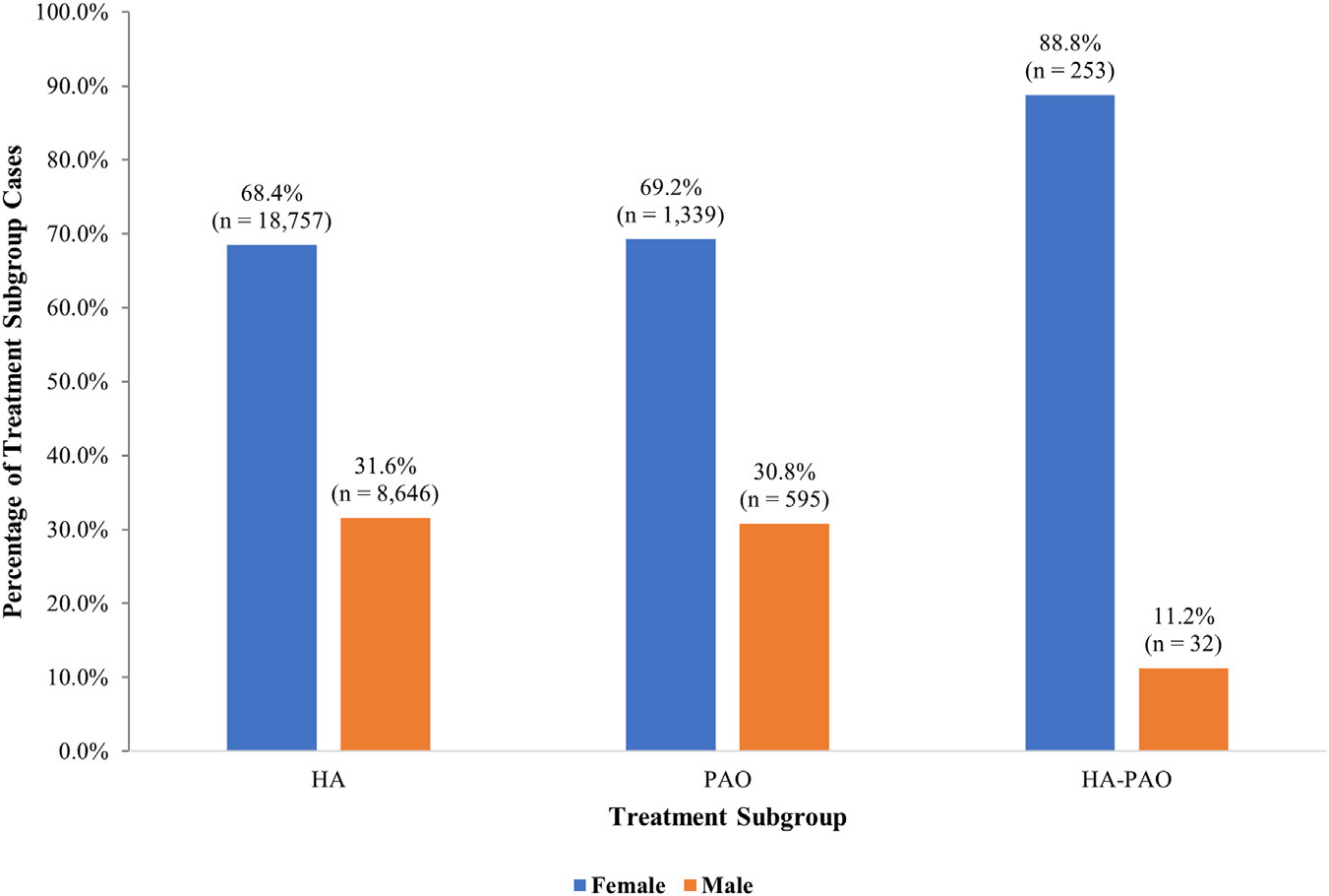
Trends by patient sex in HA, PAO, and HA-PAO for the treatment of hip dysplasia. Note the female majority. (HA, hip arthroscopy alone; HA-PAO, combined hip arthroscopy and periacetabular osteotomy; PAO, periacetabular osteotomy alone.)

HA-PAO was performed in 285 patients: there was a 4.6× increase from 2015 to 2016 and a peak of 89 cases in 2021 (Fig 4). Within this group, the majority were performed in patients aged 10 to 19 years (34.4%) and 20 to 29 years (30.9%), with only 0.7% of cases performed in the 50- to 59-year age group (Fig 2). Across all 3 cohorts, most operations were performed in female patients: 18,757 (68.4%) HA, 1,339 (69.2%) PAO, and 253 (88.8%) HA-PAO (Fig 5).

**Fig 6:**
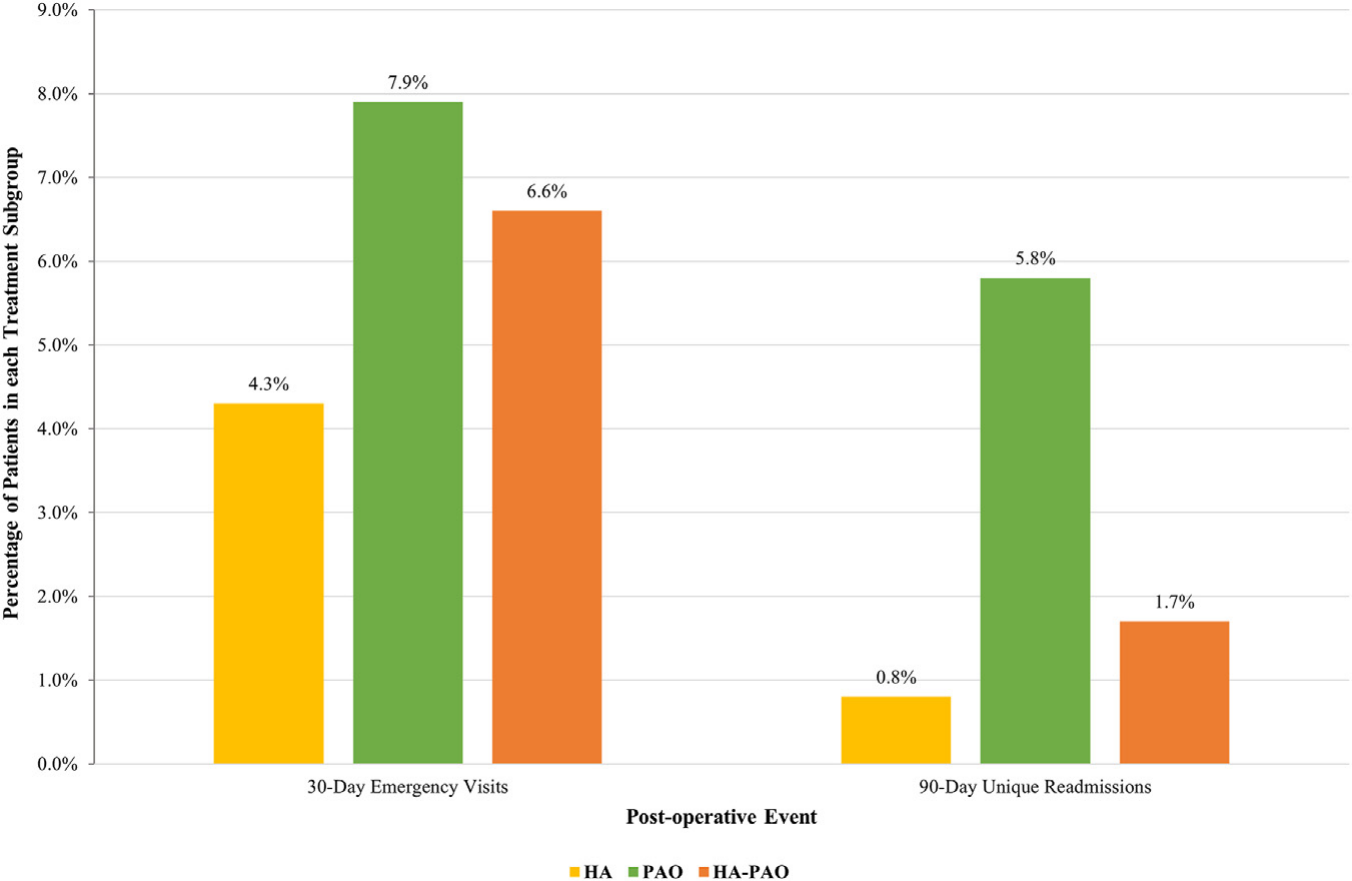
Rates of emergency visits and readmissions for HA, PAO, and HA-PAO for the treatment of hip dysplasia. Note that for readmissions, each patient is counted once. (HA, hip arthroscopy alone; HA-PAO, combined hip arthroscopy and periacetabular osteotomy; PAO, periacetabular osteotomy alone.)

The PAO cohort had the greatest percentage of 30-day postoperative emergency visits (7.9%), compared with HA-PAO (6.6%) and HA (4.3%) (Fig 6). Primary diagnoses associated with emergency visits included pain and infection. For patients who received HA, 13.2% of emergency visits were because of pain and 1.2% were because of infection; for patients who received PAO, 13.2% of emergency visits were likewise the result of pain. The diagnoses of infection in patients who received PAO and infection and pain in patients who received HA-PAO were associated with <11 emergency visits, and a percentage could not be calculated. The PAO cohort also had the greatest percentage of unique patient readmissions to the hospital within 90 days of operation (5.8%), compared with HA-PAO (1.7%) and HA (0.8%) (Fig 6). The primary surgical cause of readmission was infection in every group. For PAO, 21.6% of readmissions were caused by infection; for HA, it was 9.4%.

**Fig 7:**
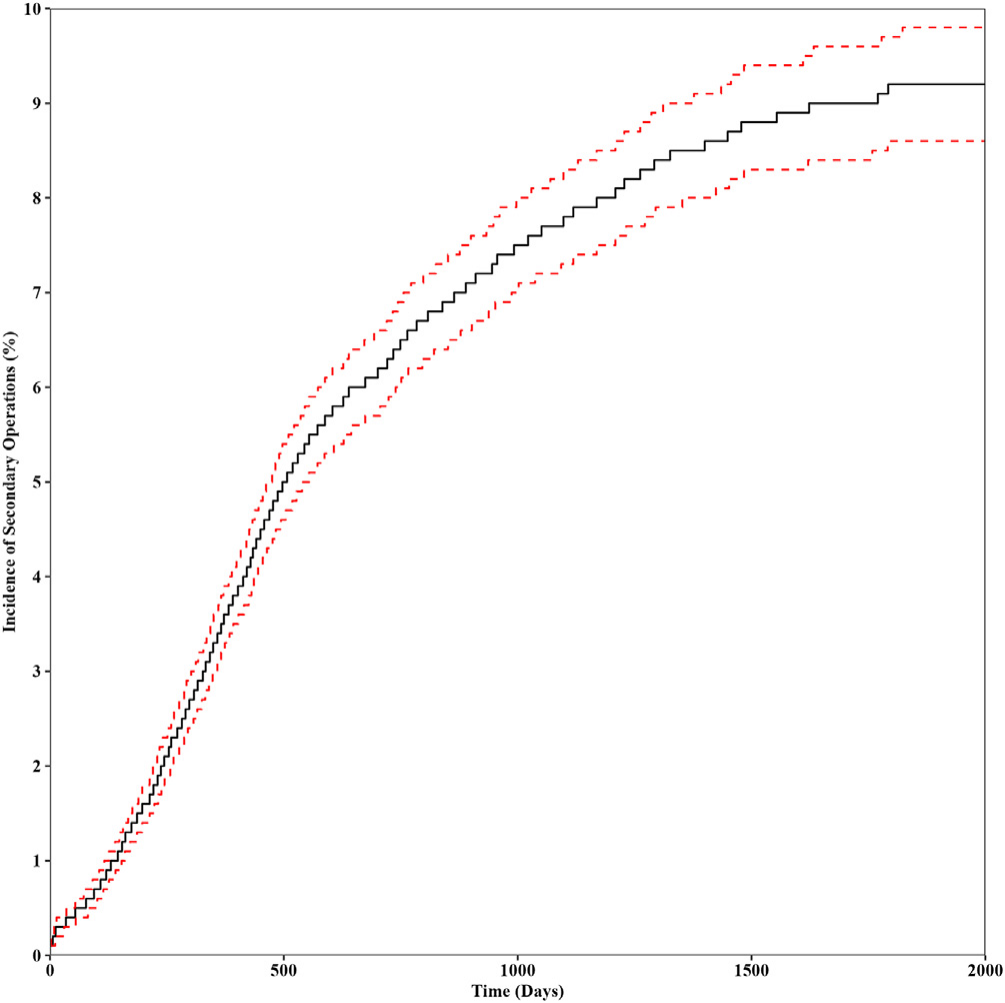
Five-year incidence of ipsilateral secondary operations in patients undergoing HA for the treatment of hip dysplasia. Secondary operations include revision hip arthroscopy, periacetabular osteotomy, or conversion to total hip arthroplasty. Data shown as the Kaplan-Meier estimate of incidence (black) with 95% confidence intervals (red). (HA, hip arthroscopy alone.)

**Fig 8:**
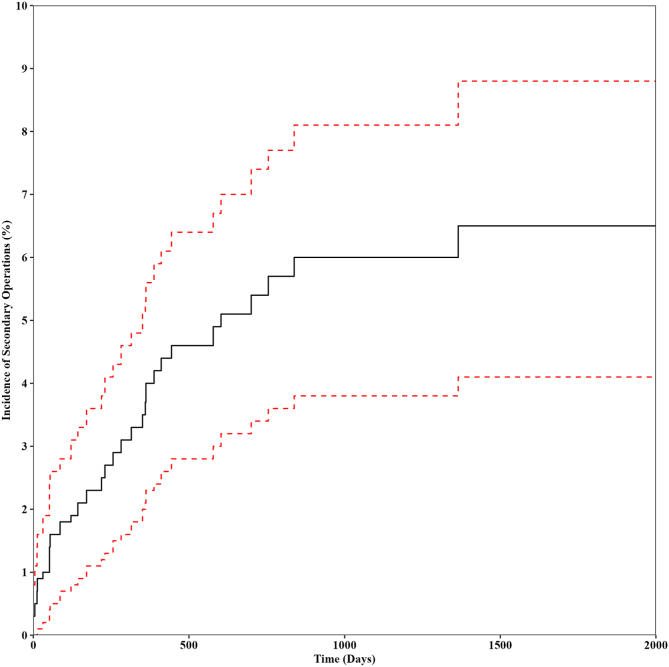
Five-year incidence of ipsilateral secondary operations in patients undergoing PAO for the treatment of hip dysplasia. Secondary operations include revision periacetabular osteotomy, hip arthroscopy, or conversion to total hip arthroplasty. Data shown as the Kaplan-Meier estimate of incidence (black) with 95% confidence intervals (red). (PAO, periacetabular osteotomy alone.)

The 5-year incidence of ipsilateral secondary operations, which include revision HA, PAO, or conversion to THA was 9.1% (95% confidence interval [CI] 8.6%-9.7%). In the HA group, there was a 9.2% (95% CI 8.6%-9.8%) reoperation rate (Fig 7), compared with a 6.5% (95% CI 4.1%-8.8%) reoperation rate in the PAO group (Fig 8). Combining HA with PAO resulted in so few secondary operations that Kaplan-Meier survival analysis was not feasible. For HA, 58.7% of the secondary procedures were revision HA. For PAO, 61.5% were revision PAOs. HA had a greater 5-year incidence of conversion to THA when compared with PAO (3.8% vs 0.7%) (Table 1).

**Table 1:** Incidence of Ipsilateral Secondary Operations After HA, PAO, and HA-PAO for the Treatment of Hip Dysplasia

Secondary Surgery	HA	PAO	HA-PAO
Any secondary surgery	9.2%	6.5%	0.0%
Hip arthroscopy	5.4%	1.8%	0.0%
Periacetabular osteotomy	0.3%	4.0%	0.0%
Total hip arthroplasty	3.8%	0.7%	–

NOTE. The rate of conversion to total hip arthroplasty was insufficient for Kaplan-Meier survival analysis in the HA-PAO cohort.

HA, hip arthroscopy alone; HA-PAO, combined hip arthroscopy and periacetabular osteotomy. PAO, periacetabular osteotomy alone.

## Discussion

In the present study, combined HA-PAO and HA alone procedures increased continuously from 2010 through 2021; PAO alone peaked in 2019 and declined thereafter, which may indicate that this procedure is more recently being combined with HA. PAO and HA-PAO were performed at greater rates in adolescent patients, compared with older patients for HA. Several factors may account for this: first, rate of labral and other soft-tissue injury is lowest in adolescents; second, this cohort is most often treated by a pediatric orthopaedic surgeon, who may not be trained in hip arthroscopy, and who may not have access to such a surgeon in an independent children’s hospital; third, favorable outcomes of PAO decline with age.^35^^,^^36^ Although all procedure types had low rates of 5-year secondary operations, the rate was lowest among patients who underwent HA-PAO, demonstrating that this combination addresses both the soft-tissue injury—such as labral tear and cartilage delamination—as well as the underlying osseous deformity.^26^^,^^30^ HA-PAO thus represents a comprehensive approach that treats soft-tissue injury from pressure concentration and edge loading caused by acetabular insufficiency in classic developmental dysplasia of the hip, as well as excessive force caused by malalignment in femoroacetabular impingement (FAI).^37^, ^38^, ^39^ A similar pattern was observed with readmission rates and emergency visits: patients undergoing HA-PAO experienced fewer events compared with PAO. These results suggest an evolution to HA-PAO as the treatment for HD, with improved outcomes. Having said this, because a statistical analysis was not feasible, given the small number of events for HA-PAO, we cannot comment on whether differences between groups are significant.

The annual rates of HA and HA-PAO are different compared with PAO. HA increased annually from 2010 to 2021, without evidence of a plateau; this is consistent with previous literature.^40^, ^41^, ^42^ The proportion of members of the American Board of Orthopaedic Surgery performing HA is increasing, and the majority of these surgeons are sports-trained.^43^ Increasing HA may be expected based on the observation that more surgeons will perform more operations.^43^^,^^44^ Increasing rates of HA may have several other causes, including broader training of surgeons in the procedure, patients beyond adolescence accessing a sports surgeon first, a conflation of degeneration with injury, and the siloing of services that introduce friction to communication and collaboration between surgeons of different training. Moreover, recent literature suggests that patients with borderline HD undergoing HA for the treatment of labral pathology or FAI experience favorable short- and longer-term outcomes.^24^^,^^45^, ^46^, ^47^, ^48^ Potential greater acceptance of HA in this patient population may be an additional contributor to the increasing frequency of HA, given that patients could not be categorized by severity of HD and were included as a single group in this study. Conversely, we found that PAO alone began to decline after 2019. Taken together with our finding that the rate of HA-PAO began to rise after 2016, this suggests that we are at an inflection point, wherein the combined operation is becoming the preferred surgical approach.

Patients who underwent HA-PAO and PAO were younger than patients who underwent HA; most patients in the former 2 groups were younger than 30 years of age, whereas the greatest rates of HA were in patients between the ages of 40 and 49 years. That patients who underwent PAO and HA-PAO tended to be younger is not surprising, as older patients are considered not ideal for osteotomy, which is a greater stressor, requires a more arduous recovery, and has potentially bigger complications (such as pelvic nonunion).^49^^,^^50^ A retrospective study of patients who underwent PAO with minimum 10-year follow-up found increasing age to be a risk factor for composite failure on univariate analysis, whereas multivariate analysis in a separate retrospective study of patients with dysplasia similarly found increasing age to be associated with increased risk of stress fracture after PAO.^51^^,^^52^ Our findings are consistent with a previous systematic review that showed a mean age of 25.1 years among patients who received PAO and a retrospective study that showed both an increasing procedural rate and greatest procedural incidence among patients aged 40 to 49 years who received HA.^40^^,^^53^ Although HA-PAO has been reported previously, the cross-sectional data from our study add novel information to the literature.^28^^,^^30^^,^^39^^,^^54^

Postoperative readmissions and emergency visits differed among the 3 cohorts, with infection and pain representing the most common procedure-related causes of each. Patients undergoing HA had the lowest rates of both readmissions and emergency visits, followed by HA-PAO, and lastly PAO. The lower rates for patients who received HA are expected and are consistent with the literature, as the procedure is shorter in duration and is performed through a smaller incision with less dissection.^55^ Although the greater readmissions and emergency visits were expected in PAO, an unexpected finding was that patients who received HA-PAO had intermediate rates for both readmissions and emergency visits.^56^ This may suggest a protective effect of lavaging the joint and surrounding soft tissues during HA against infection during the PAO that follows.^57^, ^58^, ^59^ Furthermore, it reduces the concern of soft-tissue swelling and anatomic distortion from fluid extravasation in combining the procedures.^39^^,^^60^ Lower complication rates in HA-PAO also may reflect a selection bias: patients who undergo combined procedures may be better informed and thereby more attentive to their treatment, including preoperative preparation and postoperative protocol. A recent study examining health information-seeking behavior of individuals on web-based Q&A sites found that increasing disease complexity was associated with increased questions relating to treatment; given that HA-PAO is a more complex procedure than HA or PAO alone, these patients might similarly seek more information about their treatment plan, from preoperative to operative to postoperative.^61^ Finally, it has been shown that multidisciplinary patient care teams improve patient outcomes, including every aspect of the treatment protocol at multiple points of contact via multiple levels of provider,^62^^,^^63^ whereas dedicated surgical teams consisting of individuals working together over time have a similar benefit.^64^ As such, patients undergoing combined HA-PAO may benefit from multiple points of contact with a multidisciplinary team, in addition to management by a surgical team (i.e., an arthroscopist and a PAO specialist) that has optimized the combined procedure over time.

Rates of reoperation differed among the 3 cohorts. Patients undergoing HA had the most ipsilateral secondary operations. Our finding of an overall 5-year reoperation rate of 9.2% is greater than in the literature because we focused on HD, which may be less responsive to HA because it is broader than a more focused diagnosis such as FAI or labral tear—conditions which HA is adept at treating.^34^^,^^65^, ^66^, ^67^, ^68^, ^69^, ^70^, ^71^ Compared with HA, patients who underwent PAO had a lower 5-year secondary surgery incidence of 6.5%, with 0.7% of patients converting to THA. Our rate of non-THA secondary operations in patients who underwent PAO (5.8%) is similar to what has been reported previously.^72^ Although our THA conversion rate resembles shorter-term studies in the literature, it is significantly lower as follow-up continues to 10 years and beyond, which is outside our tracking period.^72^, ^73^, ^74^ The greater secondary operation rates in patients who underwent HA are likely caused by the fact that it does not address the underlying osseous deformity that is the principal cause of joint injury and degeneration.^2^^,^^75^ By contrast, HA-PAO resulted in so few secondary operations that Kaplan-Meier survival analysis was not feasible. This reflects the fact that the underlying primary problem—osseous deformity—is addressed by osteotomy, and its secondary consequences—including labrum tear and CAM lesion—are addressed by arthroscopy.^18^^,^^29^^,^^30^^,^^39^^,^^54^ The finding may support a movement to HA-PAO as a more complete standard of care. As the rate of HA-PAO continues to increase, future studies with larger sample sizes can be done to further investigate differences among the procedures evaluated in this study. In addition, given the relative recency at which HA-PAO has begun to be performed, longer follow-up is needed to determine whether combining the procedures improves long-term hip survival.

### Limitations

There are limitations to the present study. First, it is a retrospective study using the PearlDiver administrative database. As such, accurate results depend on accurate and quality billing codes, as well as proper coding (as opposed to miscoding or noncoding) by providers. Diagnostic codes vary in specificity: for example, although CPT codes exist for “hip arthroscopy,” there are no such codes for “periacetabular osteotomy,” requiring instead a basis in previous literature on the subject as well as multiple data runs to maximize the accuracy of the sample. The same is true for the diagnosis of “hip dysplasia,” which has no specific associated diagnostic codes. Diagnosis codes do not account for the severity of dysplasia, which informs procedure selection. In addition, since PearlDiver is a large deidentified database, it was impossible to determine individual patient-specific indications for surgery or surgeon-specific details (e.g., whether HA was performed by a subspeciality-trained arthroscopist), as well as whether readmitted patients required treatment. Because this is a large database study, involvement of multiple surgeons may confound our results as the result of differing surgeon techniques. Radiographic measurements for each patient are also not available in PearlDiver and could not be assessed. Moreover, coverage may be limited to certain insurance plans and thereby not fully represent the population. In the 5-year Kaplan-Meier analysis, some patients did not have follow-up for all 5 years (e.g., because of lack of medical follow-up, or because the end date of the study was less than 5 years after the index procedure), which may underestimate reoperations. Because we examined 5-year secondary event incidence using a Kaplan-Meier time-to-event analysis, we were unable to provide a statistical comparison of groups. This is because the traditional odds ratios reported in large database studies such as this one are inappropriate for time-to-event data. Moreover, a log-rank test could also not be performed because of a limitation of the PearlDiver software when working with lateralized data. Traditional measures for comparing surgical methods, such as Clinical Improvement Scales, are also not available in PearlDiver. As such, we chose to analyze data for each treatment and evaluate secondary event incidence rather than conduct statistical comparisons. Finally, the 3 cohorts in our study vary widely in size, which affects comparisons: for example, we could not perform Kaplan-Meier survival analysis for the relatively small number of patients undergoing HA combined with PAO. It cannot be stated whether differences between groups are significant, as this study examined overall trends and outcomes for each procedure type over the study period and a statistical analysis was not performed.

## Conclusions

HA is more frequently performed than PAO for HD. HA-PAO is increasing at the greatest rate, demonstrating fewer complications and reoperations.

## Disclosures

The authors declare the following financial interests/personal relationships which may be considered as potential competing interests: A.L.Z. reports consulting or advisory for DePuy Mitek and Stryker Corporation and editorial board member for the journals *American Journal of Sports Medicine* and *Arthroscopy*. All other authors (J.S., K.F., S.E. W., I.S., M.D.) declare that they have no known competing financial interests or personal relationships that could have appeared to influence the work reported in this paper.

## Figures and Tables

**Appendix Table 1:** ICD-9, ICD-10, and CPT Codes Queried for Patient Inclusion and Secondary Surgery

Description	Codes Queried
Nonlateralized cohort codes	
Hip Dysplasia	
ICD-10-D-M25351	Other instability, right hip
ICD-10-D-M25352	Other instability, left hip
ICD-10-D-M25359	Other instability, unspecified hip
ICD-10-D-M25851	Other specified joint disorders, right hip
ICD-10-D-M25852	Other specified joint disorders, left hip
ICD-10-D-M25859	Other specified joint disorders, unspecified hip
ICD-10-D-Q6500	Congenital dislocation of unspecified hip, unilateral
ICD-10-D-Q6501	Congenital dislocation of right hip, unilateral
ICD-10-D-Q6502	Congenital dislocation of left hip, unilateral
ICD-10-D-Q651	Congenital dislocation of hip, bilateral
ICD-10-D-Q652	Congenital dislocation of hip, unspecified
ICD-10-D-Q6530	Congenital partial dislocation of unspecified hip, unilateral
ICD-10-D-Q6531	Congenital partial dislocation of right hip, unilateral
ICD-10-D-Q6532	Congenital partial dislocation of left hip, unilateral
ICD-10-D-Q654	Congenital partial dislocation of hip, bilateral
ICD-10-D-Q655	Congenital partial dislocation of hip, unspecified
ICD-10-D-Q656	Congenital unstable hip
ICD-10-D-Q6581	Congenital coxa valga
ICD-10-D-Q6589	Other specified congenital deformities of hip
ICD-10-D-Q659	Congenital deformity of hip, unspecified
ICD-9-D-71985	Other specified disorders of joint, pelvic region and thigh
ICD-9-D-75430	Congenital dislocation of hip, unilateral
ICD-9-D-75431	Congenital dislocation of hip, bilateral
ICD-9-D-75432	Congenital subluxation of hip, unilateral
ICD-9-D-75433	Congenital subluxation of hip, bilateral
ICD-9-D-75561	Coxa valga, congenital
ICD-9-D-75563	Other congenital deformity of hip (joint)
PAO	
CPT-27146	Osteotomy, iliac, acetabular or innominate bone
CPT-27147	Osteotomy with open reduction
ICD-10-P-0QS204Z	Reposition Right Pelvic Bone with Internal Fixation Device, Open Approach
ICD-10-P-0QS304Z	Reposition Left Pelvic Bone with Internal Fixation Device, Open Approach
ICD-10-P-0QS404Z	Reposition Right Acetabulum with Internal Fixation Device, Open Approach
ICD-10-P-0QS504Z	Reposition Left Acetabulum with Internal Fixation Device, Open Approach
ICD-10-P-0Q820ZZ	Division of Right Pelvic Bone, Open Approach
ICD-10-P-0Q830ZZ	Division of Left Pelvic Bone, Open Approach
ICD-10-P-0Q840ZZ	Division of Right Acetabulum, Open Approach
ICD-10-P-0Q850ZZ	Division of Left Acetabulum, Open Approach
ICD-10-P-0SS904Z	Reposition Right Hip Joint with Internal Fixation Device, Open Approach
ICD-10-P-0SSB04Z	Reposition Left Hip Joint with Internal Fixation Device, Open Approach
ICD-9-P-7985	Open reduction of dislocation of hip
Hip arthroscopy	
CPT-29860	Hip arthroscopy, diagnostic with or without biopsy
CPT-29862	Hip arthroscopy, chondroplasty, abrasion arthroplasty and/or resection of labrum
CPT-29863	Hip arthroscopy, synovectomy
CPT-29914	Hip arthroscopy, femoroplasty
CPT-29915	Hip arthroscopy, osteoplasty acetabulum
CPT-29916	Hip arthroscopy, labral repair
Total hip arthroplasty	
CPT-27130	Arthroplasty, acetabular and proximal femoral prosthetic replacement
CPT-27132	Conversion of previous hip surgery to total hip arthroplasty
Lateralized cohort codes	
Hip dysplasia (right)	
ICD-10-D-M25351	Other instability, right hip
ICD-10-D-M25851	Other specified joint disorders, right hip
ICD-10-D-Q6501	Congenital dislocation of right hip, unilateral
ICD-10-D-Q6531	Congenital partial dislocation of right hip, unilateral
Hip dysplasia (left)	
ICD-10-D-M25352	Other instability, left hip
ICD-10-D-M25852	Other specified joint disorders, left hip
ICD-10-D-Q6502	Congenital dislocation of left hip, unilateral
ICD-10-D-Q6532	Congenital partial dislocation of left hip, unilateral
Arthroscopy-related diagnosis codes (right)∗	
ICD-10-D-M25351	Other instability, right hip
ICD-10-D-M25851	Other specified joint disorders, right hip
ICD-10-D-Q6501	Congenital dislocation of right hip, unilateral
ICD-10-D-Q6531	Congenital partial dislocation of right hip, unilateral
ICD-10-D-M24151	Other articular cartilage disorders, right hip
ICD-10-D-M24851	Other specific joint derangements of right hip, not elsewhere classified
ICD-10-D-S73191A	Other sprain of right hip, initial encounter
ICD-10-D-S73191D	Other sprain of right hip, subsequent encounter
ICD-10-D-S73191S	Other sprain of right hip, sequelae
Arthroscopy-related diagnosis codes (left)∗	
ICD-10-D-M25352	Other instability, left hip
ICD-10-D-M25852	Other specified joint disorders, left hip
ICD-10-D-Q6502	Congenital dislocation of left hip, unilateral
ICD-10-D-Q6532	Congenital partial dislocation of left hip, unilateral
ICD-10-D-M24152	Other articular cartilage disorders, left hip
ICD-10-D-M24852	Other specific joint derangements of left hip, not elsewhere classified
ICD-10-D-S73192A	Other sprain of left hip, initial encounter
ICD-10-D-S73192D	Other sprain of left hip, subsequent encounter
ICD-10-D-S73192S	Other sprain of left hip, sequelae
PAO-related diagnosis codes (right)∗	
ICD-10-D-M25351	Other instability, right hip
ICD-10-D-M25851	Other specified joint disorders, right hip
ICD-10-D-Q6501	Congenital dislocation of right hip, unilateral
ICD-10-D-Q6531	Congenital partial dislocation of right hip, unilateral
ICD-10-D-M93251	Osteochondritis dissecans, right hip
ICD-10-D-S73034A	Other anterior dislocation of right hip, initial encounter
ICD-10-D-M24151	Other articular cartilage disorders, right hip
ICD-10-D-M24851	Other specific joint derangements of right hip, not elsewhere classified
ICD-10-D-M89251	Other disorders of bone development and growth, right femur
ICD-10-D-M21851	Other specified acquired deformities of right thigh
ICD-10-D-S73191A	Other sprain of right hip, initial encounter
ICD-10-D-M25551	Pain in right hip
ICD-10-D-M24351	Pathologic dislocation of right hip, not elsewhere classified
ICD-10-D-S73014A	Posterior dislocation of right hip, initial encounter
ICD-10-D-S73011A	Posterior subluxation of right hip, initial encounter
ICD-10-D-M24451	Recurrent dislocation, right hip
ICD-10-D-M21751	Unequal limb length (acquired), right femur
ICD-10-D-M1631	Unilateral osteoarthritis resulting from hip dysplasia, right hip
ICD-10-D-M21951	Unspecified acquired deformity of right thigh
ICD-10-D-S73004A	Unspecified dislocation of right hip, initial encounter
ICD-10-D-S73004D	Unspecified dislocation of right hip, subsequent encounter
ICD-10-D-S73001A	Unspecified subluxation of right hip, initial encounter
PAO-related diagnosis codes (left)∗	
ICD-10-D-M25352	Other instability, left hip
ICD-10-D-M25852	Other specified joint disorders, left hip
ICD-10-D-Q6502	Congenital dislocation of left hip, unilateral
ICD-10-D-Q6532	Congenital partial dislocation of left hip, unilateral
ICD-10-D-M93252	Osteochondritis dissecans, left hip
ICD-10-D-S73035A	Other anterior dislocation of left hip, initial encounter
ICD-10-D-M24152	Other articular cartilage disorders, left hip
ICD-10-D-M24852	Other specific joint derangements of left hip, not elsewhere classified
ICD-10-D-M89252	Other disorders of bone development and growth, left femur
ICD-10-D-M21852	Other specified acquired deformities of left thigh
ICD-10-D-S73192A	Other sprain of left hip, initial encounter
ICD-10-D-M25552	Pain in left hip
ICD-10-D-M24352	Pathologic dislocation of left hip, not elsewhere classified
ICD-10-D-S73015A	Posterior dislocation of left hip, initial encounter
ICD-10-D-S73012A	Posterior subluxation of left hip, initial encounter
ICD-10-D-M24452	Recurrent dislocation, left hip
ICD-10-D-M21752	Unequal limb length (acquired), left femur
ICD-10-D-M1632	Unilateral osteoarthritis resulting from hip dysplasia, left hip
ICD-10-D-M21952	Unspecified acquired deformity of left thigh
ICD-10-D-S73005A	Unspecified dislocation of left hip, initial encounter
ICD-10-D-S73005D	Unspecified dislocation of left hip, subsequent encounter
ICD-10-D-S73002A	Unspecified subluxation of left hip, initial encounter

NOTE. Codes reported with decimal points removed, per PearlDiver coding format.

CPT, Current Procedural Terminology; ICD, *International Classification of Diseases*; PAO, periacetabular osteotomy.

∗ Diagnosis codes were linked to ICD or CPT codes corresponding to PAO and hip arthroscopy during the lateralization process. PAO and arthroscopy-related diagnosis codes were additionally used to lateralize total hip arthroplasty procedures, as all arthroplasty patients underwent primary PAO or hip arthroscopy beforehand.

## Supplementary Data

ICMJE author disclosure forms

ICMJE author disclosure forms

ICMJE author disclosure forms

ICMJE author disclosure forms

ICMJE author disclosure forms

ICMJE author disclosure forms
