# Supplementary material for: The Use of Combined Hip Arthroscopy and Periacetabular Osteotomy for Hip Dysplasia Is Increasing and Has Low Complication Rates
Source: Arthrosc Sports Med Rehabil. 2024 Mar 26;6(3):100929. doi: 10.1016/j.asmr.2024.100929 (PMC11240039; doi:10.1016/j.asmr.2024.100929)
Supplement: ICMJE author disclosure forms [file mmc6.docx]

**Declaration of interests**
 
☐ The authors declare that they have no known competing financial interests or personal relationships that could have appeared to influence the work reported in this paper.
 
☒ The authors declare the following financial interests/personal relationships which may be considered as potential competing interests:

| Alan L. Zhang reports a relationship with DePuy Mitek Inc that includes: consulting or advisory. Alan L. Zhang reports a relationship with Stryker Corporation that includes: consulting or advisory. Alan L. Zhang–Editorial Board Member for the journals: American Journal of Sports Medicine, Arthroscopy. If there are other authors, they declare that they have no known competing financial interests or personal relationships that could have appeared to influence the work reported in this paper. |
| --- |
